# Association between water insecurity and antiretroviral therapy adherence among pregnant and postpartum women in Greater Accra region of Ghana

**DOI:** 10.1371/journal.pgph.0002747

**Published:** 2024-01-08

**Authors:** Jerry John Nutor, Jaffer Okiring, Isaac Yeboah, Rachel G. A. Thompson, Pascal Agbadi, Edward Kwabena Ameyaw, Monica Getahun, Wisdom Agbadi, Thomas J. Hoffmann, Sheri D. Weiser

**Affiliations:** 1 Department of Family Health Care Nursing, School of Nursing, University of California San Francisco, San Francisco, California, United States of America; 2 Infectious Diseases Research Collaboration, Kampala, Uganda; 3 Institute of Work Employment and Society, University of Professional Studies, Accra, Ghana; 4 Language Center, College of Humanities, University of Ghana, Accra, Ghana; 5 Africa Interdisciplinary Research Institute, Accra, Ghana; 6 Department of Sociology and Social Science Policy, Lingnan University, Hong Kong, China; 7 Institute of Policy Studies and School of Graduate Studies, Lingnan University, Hong Kong, China; 8 Institute for Global Health Sciences, University of California, San Francisco, San Francisco, California, United States of America; 9 Push Aid Africa, Accra, Ghana; 10 Department of Epidemiology and Biostatistics, and Office of Research, School of Nursing, University of California, San Francisco, San Francisco, California, United States of America; 11 Division of HIV, Infectious Diseases and Global Medicine, School of Medicine, University of California, San Francisco, San Francisco California, United States of America; North Carolina State University, UNITED STATES

## Abstract

**Background:**

Adherence to antiretroviral therapy (ART) can substantially reduce morbidity and mortality among women living with HIV (WLWH) and prevent vertical transmission of HIV. However, in sub-Saharan Africa (SSA), more than 50% of new mothers discontinue ART and HIV care after childbirth. The role of water insecurity (WI) in ART adherence is not well-explored. We examined the relationship between WI and ART adherence among pregnant and postpartum WLWH in Greater Accra region of Ghana.

**Methods:**

Using a cross-sectional survey, we recruited 176 pregnant and postpartum WLWH on ART across 11 health facilities. We examined the association between WI (measured using the Household Water Insecurity Experience Scale, and categorized as moderate and severe WI compard to low WI) and poor ART adherence (defined as scoring a below average observed CASE index score). Bivariate analysis was performed using chi-square test followed by multivariate logistic regression models. We included all variables with p-values less than 0.20 in the multivariate analysis.

**Results:**

Most (79.5%) of the pregnant and postpartum WLWH enrolled on ART, were urban residents. Over 2/3 were aged 30 years and older. Overall, 33.5% of respondents had poor ART adherence. Proportion of poor ART adherence was 19.4% among those with low WI, 44.4% in those with moderate WI, and 40.0% among those with high WI. Respondents with moderate household water insecurity had a greater odds of reporting poor ART adherence, as compared to those with low water insecurity (adjusted Odds ratio (aOR) = 2.76, 95%CI: 1.14–6.66, p = 0.024), even after adjusting for food insecurity. Similarly, respondents with high WI had a greater odds of reporting poor ART adherence, as compared to those with low water insecurity (aOR = 1.49, 95%CI: 0.50–4.48, p = 0.479), even after adjusting for food insecurity.

**Conclusion:**

Water insecurity is prevalent among pregnant and postpartum WLWH and is a significant risk factor for poor ART adherence. Governments and other stakeholders working in HIV care provision should prioritize water security programming for WLWH along the HIV care continuum.

## Introduction

Approximately 38.4 million people worldwide are living with HIV; of those, 25.6 million people (67%) reside in sub-Saharan Africa (SSA), including in Ghana [1]. Women represent more than half of the people living with HIV. Further, infants born to women living with HIV (WLWH) are more susceptible to HIV through vertical transmission during pregnancy, childbirth, or breastfeeding [2, 3].

The advent of antiretroviral therapy (ART) has been associated with reduced HIV/AIDS related morbidity, AIDS related deaths, secondary transmission, improved quality of life and effective management of HIV [4, 5]. Yet, despite increased access to ARTs, only 75% of the global and 67% of people living with HIV in SSA had access to ART in 2021, making it improbable to meet the 95-95-95 targets by 2030 [6]. In 2019, among the 219,986 women with HIV in Ghana ART coverage was even lower at 54%. In the same year, of the 15,599 pregnant women with HIV, only an estimated 75% received ART through prevention of mother-to-child transmission services [7]. Yet, achieving optimal suppression and prevention of onward transmission requires high levels of ART adherence [8].

Studies on ART adherence demonstrate large variation in adherence between and within regions and countries of the world, largely due to context-specific socio-cultural factors [9]. For instance, studies have reported 92% ART adherence rate in Nigeria, [10] 75% in Uganda, [11] 69% in South Africa, [12] and 78.4% in Togo [13]. Studies of pregnant and postpartum women in Greater Accra and Tema Metropolitan regions in Ghana showed adherence rates of 26.7% and 85.1%, respectively [9, 14]. Another study of reproductive age women in Kumasi in the Ashanti Region of Ghana reported an ART uptake of 27% [15]. This wide disparity in ART adherence across regions is reflective of the contextual and geographic differences that impact care access.

Studies have also highlighted the role of food insecurity (FI) in ART adherence in SSA [16–18]. There are several pathways through which food insecurity (WI) may impact ART adherence, including perceived side effects of taking ART without sufficient food as well as the challenges of managing FI in the context of increased appetite while taking ART. However, very few studies have explored the relationship between WI and ART adherence. The studies to date, have shown how lack of access to clean water, insufficient water, and low water quality might increase poor economic conditions of households and communities which are important pathways towards lowered adherence and decreased care engagement [19–26]. Twenty six percent (26%) of the global population does not have access to clean water as compared to 40% of the population in sub-Sharan Africa [27]. Individuals receiving ART may forgo appointments and their livelihoods in search of clean water, which may in turn exacerbate their poor economic conditions and geographical factors such as urban vs rural residence [23, 24, 28, 29]. Additionally, water insecurity is associated with stress and poor mental health, and may subsequently limit people living with HIV ability to adhere to ART and engage in care [30, 31].

WI is a highly gendered experience in SSA, women are primarily responsible for fetching water and ensuring adequate water for household members [32, 33]. Women are vulnerable to HIV due to exposures in pursuit of water and other resources. During water scarcity, women may also need to rely on transactional sex to survive [34, 35]. For instance, a study in Lesotho found that women residing in water insecure communities had higher odds of risky sexual behaviors including early sexual debut and transactional sex, known pathways to increased HIV transmission [36]. For pregnant and postpartum women, the demand for water increases regardless of HIV status [37]. Women living with HIV may also have a significantly higher demand for household water due to the need to maintain adequate hydration while on ART and/or managing unpleasant side effects such as nausea, vomiting and diarrhea [20, 38, 39].

We examined the associations between WI and ART adherence among pregnant and postpartum WLWH in Greater Accra. We hypothesize that household water insecurity would be associated with poor ART adherence, even after accounting for FI and other confounders such as age, partner’s known HIV status, and fuel type mainly used. This study addresses an important research gap on the relationship between HIV and WI, especially among women living with HIV.

## Materials and methods

### Study design and setting

This cross-sectional study of pregnant and postpartum women receiving ART was conducted in 11 hospitals and health centers designated for pre- and post-natal care in Greater Accra, Ghana, a high HIV prevalence region [40]. This survey was conducted between March and May 2022.

### Sampling procedures and recruitment

We used convenience sampling to recruit N = 176 pregnant and postpartum women living with HIV at their pre- or post-natal visits to the health facility in consultation with their clinical care team during the study period. Pregnant or postpartum women were included in the study if they were on ART for at least 2 months, 18 years or older, willing to provide written informed consent, and spoke English or one of the local languages. We excluded women who were: 1) mothers with serious medical or psychiatric conditions that would limit participation in study, and 2) with a medical history of intellectual impairment, ascertained from medical records.

### Data collection instrument and management

Electronic questionnaires were designed including range checks and internal consistency checks using Research Electronic Data Capture (REDCap) online designer tool and piloted internally among 15 individuals to determine the intergrity of the data collection tool [41]. We gathered socio-demographic variables, household FI, household WI, water and sanitation infrastructure and access. We also collected additional water related expousres such as type (sachet water, pipe water) and time use to collect water (S1 Table). We measured WI using the Household Water InSecurity Experiences (HWISE) Scale [22]. The HWISE Scales holistically measure the human experience across four domains: water availability, accessibility, use, and stability. The scale consists of 12 questions on household experience of life-disrupting water-related problems in the previous 12 months [22]. The items pertain to psychosocial distress related to water (worry, anger, shame), water-induced disruptions in hygiene practices (inadequate water for handwashing, bathing, laundering), and issues pertaining to water consumption (not having desired amounts of water to drink, having to change foods eaten, going to sleep thirsty) [22]. FI and WI were measured using the parameters presented in S1 Table. We adapted nine validated parameters from Household Food Insecurity Access Scale to measure FI [42, 43]. Similarly, twelve parameters for WI were adapted from the validated Household Water Insecurity Experience (HWISE) Scale questionnaire [22]. These parameters in S1 Table were used to generate composite scores for FI and WI, and then converted the scores into three tertiles indicating low, moderate, and high FI and WI, respectively. We also collected data on the type of toilet facilities used by the participants and their household. The question on toilet type and use include: type of toilet facility most used in household; where toilet flushes to and information on toilet facilities sharing with others. Data were collected via REdCap programmed tablets. Six trained research assistants conducted the surveys in-person, in private spaces within the facilities and in English or the local language, based on respondent preference.

### Primary outcome

The primary outcome variable was ART adherence as measured by the Center for Adherence Support Evaluation (CASE) adherence score index [44]. ART adherence was reported utilizing three main 5-point Likert scale questions: 1) how often do you feel that you have difficulty taking your HIV medications on time?; 2) on average, how many days per week would you say that you missed at least one dose of your HIV medications?; and 3) when was the last time you missed at least one dose of your HIV medications? We obtained the observed average CASE adherence score and generated a binary variable indicating a below average or not below average score. All respondents with below average score were classified as a poor CASE index, while above average score as a good CASE index.

### Sample size determination

We used a type I error of 0.025, standardized minimum detectable effect size from a standard normal distribution for adherence β = 0.26 (equivalent to odds ratio of 1.30), and simulated 100 or 150 individuals. This sample size of 150 individuals returned a statistical power of 80%. We then adjusted for 30% stopping treatment, resulting a sample size of 176 individuals. We used the proportion of participants with low WI among those with good (46.2%) and poor (22.0%) ART adherence from our study to statistical power to determine a significant difference using a formula proposed by Wang and Chow, [45] the statistical power was 87.6%.

Similarly, we also computed the minimum detectable effect (MDE) using two-sample proportions—Pearson’s chi-squared test and the normal approximation correction for continuity. The MDE odds ratio of 2.65 was obtained for our sample size of 176, power 80%, level of significance of 5%, and proportion of low WI among those with poor ART adherence of 22.0% from our study. The MDE odds ratio of 2.65 indicates that we cannot detect any effect size below 2.65 precisely from zero, even if it exists.

### Statistical analyses

Data were analysed using Stata version 14.1 (College Station, TX). We conducted descriptive statistics including frequencies and percentages for categorical variables and means (standard deviations) or medians (and interquartile range, if skewed) for continuous variables. We conducted factor analysis of the variables related to CASE, household FI and household WI to assess construct validity, and Cronbach’s alpha to assess internal consistency. Bivariate analysis were performed using chi-square tests and univariate logistic regression models to identify potential factors associated with CASE adherence scores. We included all variables with p-value <0.20 in the multivariate analysis. In the model building process, we used either district-level or facility-level random components. But in either district-level or facility-level random components, the ICC values were negligible, we considered clustering at facility-level. Therefore, associations between WI and ART adherence were estimated using multivariable logistic regression models with facility-level clustered robust standard errors The best model was selected using the Akaike Information Criterion. Cases with missing data were eliminated in the multivariate analyses. Measures of association were expressed as odds ratios and adjusted odds ratios. A two-sided p-value <0.05 was considered statistically significant. We further describe the patterns of water use among different ART adherence scores by providing proportions.

### Ethical considerations

The protocol for this study and the informed consent form were approved by the University of California San Francisco Institutional Review Board with approval number 21–35733 and Ghana Health Service Ethics Review Committee number GHS-ERC: 003/12/21. Respondents gave informed consent prior to enrollment using the approved consent forms.

## Results

### Characteristics of the study population

Overall, most respondents (79.5%) were urban residents, married or partnered (96.0%), and knew their partner’s HIV status (63.1%). Most (89.0%) had at least one child and were in monogamous unions (89.3%); over half (60.8%) had attained at least a primary education. Most (63.6%) reported using liquefied petroleum gas (LPG) or natural gas for cooking.

A moderate proportion of households 38.1% and 30.7%, reported high and moderate household WI, respectively while 35.8% reported high WI. Thirty two percent (32.4%) had moderate food insecurity (Table 1). Overall, most (67.5%) respondents scored a better ART adherence compared to 33.5% scoring poor adherence. The Cronbach’s alpha values for household WI was great (0.90), and food insecurity (0.87), indicating high internal consistency.

**Table 1:** Socio-demographic characteristics (n = 176).

Characteristics	Category	Frequency (%)
Place of residence	Peri-urban/Rural	36 (20.5)
	Urban	140 (79.5)
Age	<30 years	54 (30.7)
	30–34	64 (36.4)
	> = 35 years	56 (31.8)
	Missing	2 (1.1)
Marital status	Married/separated	98 (55.7)
	In a relationship, living/not with a partner	71 (40.3)
	Single, never married, no current partner	7 (4.0)
Partner’s HIV status known	No	65 (36.9)
	Yes	111 (63.1)
Have rival/co-wife or co-wives	Yes	18 (10.2)
	No	151 (85.8)
	Missing	7 (4.0)
Number of children	None	19 (10.8)
	1–2 children	82 (46.6)
	3 or more children	72 (40.9)
	Missing	3 (1.7)
Religion	Protestant/catholic	33 (18.8)
	Pentecostal	54 (30.7)
	Charismatic	57 (32.4)
	Others (Adventist/Muslim/..)	32 (18.1)
Education status	Less than primary	47 (26.7)
	Completed primary	60 (34.1)
	Completed O level	42 (23.9)
	Completed A level and above	27 (15.3)
Fuel type mainly used	LPG or natural gas	112 (63.6)
	Charcoal/wood	64 (36.4)
Household Food Insecurity score	Low food insecurity	63 (35.8)
	Moderate food insecurity	57 (32.4)
	High food insecurity	56 (31.8)
Household Water Insecurity score	Low water insecurity	67 (38.1)
	Moderate water insecurity	54 (30.7)
	High water insecurity	55 (31.3)

### Patterns of water use among respondents with different levels of ART adherence scores

Over two-thirds of the respondents (70.4%) reported using sachet water (a form of selling pre-filtered or sanitized water in plastic, heat sealed bags in parts of the global south, and are especially popular in Africa) as their main source of drinking water. Among these, 30.6% had poor ART adherence as compared to those with public tap/ stand pipe (47.2%) and others (25.0%). About half (56.3%) of respondents spent minutes to go get water. Among them, 33.3% had poor ART adherence compared to those with water at their premises (36.9%), and 16.7% who did not know. Adult women were reported to usually fetch water (56.8%). And among them, 37.0% had poor ART adherence compared to adult men (30.8%), women under 15 years (47.1%), men under 15 years (40.0%). Overall, most (87.4%) participants do not treat water for drinking. Among these participants who do not treat drinking water, 35.9% had poor ART adherence compared to 8.3% of those who treat drinking water, and 20% who declined to answer. More details of the distribution of water and toilet exposures by levels of ART adherence score are presented in Table 2. Unlike type of flashing system, the differences in the rest of water and toilet exposures were not statistically significant (p>0.05).

**Table 2:** Water and toilet situation among households stratified by level of ART adherence score.

Characteristics	Category	ART adherence score			
		Overall (%)	Good (column, row %)	Poor (column, row %)	p
	
Main source of drinking-water for members	Public tap/standpipe	36 (20.5)	19 (16.2, 52.8)	17 (28.8, 47.2)	0.134
	Sachet water	124 (70.4)	86 (73.5, 69.4)	38 (64.4, 30.6)	
	Others(….)	16 (9.0)	12 (10.3, 75.0)	4 (6.8, 25.0)	
Main source of water used by your household for other purposes, such as cooking and hand washing	Piped water into dwelling	40 (22.7)	26 (22.2, 65.0)	14 (23.7, 35.0)	0.736
	Piped water to yard/plot	33 (18.8)	23 (19.6, 69.7)	10 (17.0, 30.3)	
	Public tap/standpipe	62 (35.2)	38 (32.5, 61.3)	24 (40.7, 38.7)	
	Tube well/borehole	9 (5.1)	5 (4.3, 55.6)	4 (6.8, 44.4)	
	Unprotected/protected dug well	8 (4.6)	7 (6.0, 87.5)	1 (1.6, 12.5)	
	Cart with small tank/drum	17 (9.6)	13 (11.1, 76.5)	4 (6.8, 23.5)	
	Others	7 (4.0)	5 (4.3, 71.4)	2 (3.4, 28.6)	
How long does it take to go there, get water, and come back	Minutes	99 (56.3)	66 (56.4, 66.7)	33 (55.9, 33.3)	0.451
	Water on premises	65 (36.9)	41 (35.0, 63.1)	24 (40.7,36.9)	
	Don’t know	12 (6.8)	10 (8.6, 83.3)	2 (3.4, 16.7)	
Who usually goes to this source to fetch the water for your household	Adult woman	100 (56.8)	63 (53.9, 63.0)	37 (62.7, 37.0)	
	Adult man	26 (14.8)	18 (15.4, 69.2)	8 (13.6, 30.8)	0.114
	Female child (under 15 years)	17 (9.7)	9 (7.7, 52.9)	8 (13.6, 47.1)	
	Male child (under 15 years)	5 (2.8)	3 (2.5, 60.0)	2 (3.4, 40.0)	
	Don’t know	28 (15.9)	24 (20.5, 85.7)	4 (6.7, 14.3)	
Do you treat your water in any way to make it safer to drink	Yes	12 (6.9)	11 (9.4, 91.7)	1 (1.7, 8.3)	
	No	153 (87.4)	98 (83.8, 64.1)	55 (94.8, 35.9)	0.088
	Declined to answer	10 (5.7)	8 (6.8, 80.0)	2 (3.5, 20.0)	
What do you usually do to the water to make it safer to make it safer to drink	Boil (n = 176)	3 (1.7)	3 (2.6, 100.0)	0 (0.0, 0.0)	-
	Add bleach/chlorine (n = 176)	9 (5.1)	8 (6.8, 88.9)	1 (1.7,11.1)	-
	Strain it through a cloth (n = 176)	0 (0.0)	0 (0.0, 0.0)	0 (0.0, 0.0)	-
	Use water filter (n = 176)	0 (0.0)	0 (0.0, 0.0)	0 (0.0, 0.0)	-
	Solar disinfection (n = 176)	0 (0.0)	0 (0.0, 0.0)	0 (0.0, 0.0)	-
	Let it stand and settle (n = 176)	0 (0.0)	0 (0.0, 0.0)	0 (0.0, 0.0)	-
	Others (n = 176)	1 (0.6)	1 (0.9, 100.0)	0 (0.0, 0.0)	-
kind of toilet facility used most in your household	Flush/pour flush to	99 (56.3)	68 (58.1, 68.7)	31 (52.5, 31.3)	0.481
	Ventilated improved pit latrine (VIP)	77 (43.7)	49 (41.9, 63.6)	28 (47.5, 36.4)	
Type of flashing system (n = 99)	Piped sewer system	48 (48.5)	34 (50.0, 70.8)	14 (45.2, 29.2)	
	Septic tank	31 (31.3)	16 (23.5, 51.6)	15 (48.4,48.4)	0.012
	Pit latrine	12 (12.1)	12 (17.7,100.0)	0 (0.0, 0.0)	
	Others	8 (8.1)	6 (8.8, 75.0)	2 (6.4, 25.0)	
Specify where toilet flushes to (N = 77)	Pit latrine with slab	48 (62.3)	32 (65.4, 66.7)	16 (57.1, 33.3)	0.911
	Pit latrine without slab/open pit	11 (14.3)	7 (14.3, 63.6)	4 (14.3, 36.4)	
	Composting toilet	2 (2.6)	1 (2.0, 50.0)	1 (3.6, 50.0)	
	Hanging toilet/hanging latrine	1 (1.3)	1 (2.0, 100.0)	0 (0.0, 0.0)	
	No facilities or bush or field	4 (5.2)	2 (4.1, 50.0)	2 (7.1, 50.0)	
	others	11 (14.3)	6 (12.2, 54.6)	5 (17.9, 45.4)	
Do you share this facility with other households	Yes	132 (75.0)	86 (73.5, 65.2)	46 (78.0, 34.8)	0.808
	No	43 (24.4)	30 (25.6, 69.8)	13 (22.0, 30.2)	
	Unsure	1 (0.6)	1 (0.9, 100.0)	0 (0.0, 0.0)	
How many households use this toilet facility	One	31 (17.6)	19 (16.2, 61.3)	12 (20.3, 38.7)	
	More than one	126 (71.6)	81 (69.2, 64.3)	45 (76.3, 35.7)	0.072
	Unsure	19 (10.8)	17 (14.5, 89.5)	2 (3.4, 10.5)	

Column % refer to exposure prevalence by outcome group, row % refer to outcome prevalence by exposure group

A large proportion of respondents (87.4%) indicated that they do not treat water for safety. This was similar across all adherence scores: 83.8% of those with good ART adherence scores, and 94.8% of those with poor adherence scores reported the same. Further, 56.3% reported mostly using flush/pour flush toilets, while 43.7% mostly used ventilated improved pit latrines, with similar observations across adherence groups. Overall, 75.0% of the respondents shared a toilet facility with others: this pattern was also similar across ART adherence levels (S2 Table).

### Effect of water insecurity on ART adherence

Overall, 33.5% of respondents had poor ART adherence. Proportion of poor ART adherence was 19.4% among those with low WI, 44.4% in those with moderate WI, and 40.0% among those with high WI. In bivariate analyses, respondents with moderate (OR = 3.06, 95% CI: 1.32–7.09, p = 0.009), and high (OR = 2.48, 95% CI: 1.04–5.90, p = 0.040) WI had greater odds of poor ART adherence scores, as compared to those with low household WI. In multivariable analysis (adjusting for place of residence, age, Partner’s HIV status, fuel type, and FI), respondents with moderate WI had greater odds of poor ART adherence scores (aOR = 2.76, 95% CI: 1.14–6.66, p = 0.024) as compared to those with low WI. High WI was not significantly associated with poor ART adherence scores in adjusted analyses (OR = 1.49, 95% CI: 0.50–4.48, p = 0.479) (Table 3).

**Table 3:** Factors associated with poor ART adherence score (N = 174).

Characteristics	Category	Outcome present (%)	Bivariate analysis		Multivariable analysis ^b^ (N = 174)		Model adjusting FI only	
			ORs (95% CI)	p	aORs (95% CI)	p	aORs (95% CI)	p
Place of residence	Urban	52 (37.1)	Reference	-	Reference	-	-	-
	Peri-urban/Rural	7 (19.4)	0.49 (0.18–1.36)	0.171	0.44 (0.25–0.77)	0.004	-	-
Age	<30 years	25 (46.3)	Reference	-	Reference	-	-	-
	30–34	17 (25.8)	0.42 (0.19–0.93)	0.033	0.30 (0.13–0.69)	0.004	-	-
	> = 35 years	17 (30.4)	0.51 (0.23–1.15)	0.106	0.51 (0.32–0.82)	0.006	-	-
Marital status	Single, never married, no current partner	2 (28.6)	Reference	-	-	-	-	-
	Married/separated	32 (32.7)	0.80 (0.13–4.90)	0.811	-	-	-	-
	In a relationship, living/not with a partner	25 (35.2)	0.84 (0.13–5.37)	0.854	-	-	-	-
Partner’s HIV status known	No	30 (46.2)	Reference	-	Reference	-	-	-
	Yes	29 (26.1)	0.43 (0.21–0.86)	0.017	0.45 (0.24–0.85)	0.014	-	-
Have rival/co-wife or co-wives	Yes	10 (55.6)	Reference	-	-	-	-	-
	No	47 (31.1)	0.46 (0.15–1.40)	0.172	-	-	-	-
Number of children	None/refused	9 (40.9)	Reference		-	-	-	-
	1–2 children	28 (34.2)	0.77 (0.28–2.13)	0.620	-	-	-	-
	3 or more children	22 (30.6)	0.63 (0.22–1.79)	0.384	-	-	-	-
Religion	Charismatic	20 (35.1)	Reference	-	-	-	-	-
	Protestant/catholic	9 (27.3)	0.77 (0.29–2.05)	0.599	-	-	-	-
	Pentecostal	18 (33.3)	0.85 (0.36–2.00)	0.709	-	-	-	-
	Others (Adventist/Muslim/)	12 (37.5)	0.98 (0.38–2.54)	0.961	-	-	-	-
Education status	Completed primary	27 (45.0)	Reference	-	-	-	-	-
	Less than primary	12 (25.5)	0.44 (0.19–1.02)	0.057	-	-	-	-
	Completed O level	12 (28.6)	0.57 (0.23–1.40)	0.219	-	-	-	-
	Completed A level and above	8 (29.6)	0.55 (0.20–1.53)	0.253	-	-	-	-
Fuel type mainly used	LPG or natural gas	44 (39.3)	Reference	-	Reference	-	-	-
	Charcoal/wood	15 (23.4)	0.48 (0.23–1.01)	0.053	0.38 (0.14–1.02)	0.056	-	-
Household food insecurity score	Low	19 (30.2)	Reference	-	Reference	-	Reference	-
	Moderate	17 (29.8)	0.97 (0.43–2.18)	0.937	1.14 (0.48–2.68)	0.769	0.70 (0.29–1.69)	0.431
	High	23 (41.1)	1.49 (0.66–3.37)	0.334	1.69 (0.82–3.49)	0.158	1.22 (0.53–2.79)	0.635
Household water insecurity score^a^	Low	13 (19.4)	Reference	-	Reference	-	Reference	-
	Moderate	24 (44.4)	3.06 (1.32–7.09)	0.009	2.76 (1.13–6.66)	0.024	3.53 (1.50–8.29)	0.004
	High	22 (40.0)	2.48 (1.04–5.90)	0.040	1.49 (0.50–4.48)	0.479	2.79 (1.13–6.87)	0.026

^b^final multivariable model was selected using Akaike inclusion criteria and adjusted for facility-level clustered standard errors

## Discussion

In this cross-sectional study, we set out to determine the association between WI and ART adherence among pregnant and postpartum WLWH in Greater Accra. To our knowledge this is the first study which focuses on understanding the association between ART adherence and WI among pregnant and postpartum WLWH. We found that 68.8% of the respondents had moderate to high household water insecurity. Nearly, half of the respondents with average or high adherence scores experienced low household WI (46.2%), while 28.2% had high-water insecurity. In multivariable analyses, we found that respondents with moderate household WI scores had nearly three times the odds of a below average ART adherence score, as compared to those with a low household WI score. Overall, this finding suggests that WI is a potential barrier to ART adherence, as previously reported in a study conducted in Zambia [20, 46]. This finding suggests the importance of WI in ART adherence among people living with HIV, and especially among pregnant and postpartum women. Our findings are also consistent with a study of pregnant and postpartum WLWH in Zambia which found that respondents with access to water were more likely to have higher ART adherence intentions, as compared to those with limited access to water [20]. Similarly our finding is consistent with studies conducted in Botswana [23] and Kenya [24] which found that WI influence ART adherence and engagement in HIV care among people living with HIV.

Further, we found that toilet access may be associated with WI and ART adherence. Respondents with below average ART adherence scores reported higher rates of use of flush/pour (52.5%) toilets, as compared to ventilated improved pit latrines (47.5%). Those with ventilated improved pit latrines do not need water to use their toilet, suggesting that respondents with flush toilet have increased water demands in addition to water for cooking and drinking purposes. This findings suggests that, in times of limited tap water access, respondents with flush toilets might not be able to use their facilities [47]. These situation may be more challenging for pregnant and postpartum WLWH, [46] and those experiencing unpleasant side such as nausea, vomiting and diarrhea [20, 38].

The implications of this study are far-reaching, considering the vast number of people suffering from WI and the estimated 6 million of people living with HIV who are receiving ART in SSA; [48, 49] further, WI disproportionately affects pregnant and postpartum WLWH [20, 50]. More than half (62%) of our respondents were water insecure and disturbingly, most of them were highly insecure, a rate much higher than that previously reported in the general population [42, 51]. The higher rate of WI in our study population might be due to the higher demand of water among pregnant and postpartum women compared to the general population. This poses a significant challenge in the progress towards the 95-95-95 prevention and care targets of diagnosing 95% of all people living with HIV, place 95% of diagnosed people on ART, and achieving 95% viral suppression in treated people by 2030.

### Strengths and limitations

There are several limitations to our study. First, as the study was cross-sectional, it is important to note that associations are not proof of causation. Second, all measures in the current study were self-reported, which may be biased due to social desirability, particularly when the survey is administered face-to-face. However, the research data collectors were trained in building rapport and comfort with respondents, including providing options for respondents to skip uncomfortable questions. Third, our sample size was limited could not account for clustering. Further, it is important to note that our study did not control for income and other wealth indicators therefore, the observed associations may from residual confounding from socio-economic status. Finally, our study was limited to WLWH who were pregnant and postpartum in predominately urban area of Ghana, and hence results may not be generalizable to other populations.

Despite the limitations, this is a novel study and the first to examine the relationship between WI and ART adherence among WLWH in Greater Accra. Prior studies relied on other individual or structural level factors which influence ART adherence and did not capture the relationship between household WI and ART adherence. Thus, our study serves as an important foundation for future studies in this area. Future studies should more closely examine the relationship between household WI and ART adherence utilizing and supplementing self-reported measures with objective adherence markers such as hair [52] or urine [53].

### Conclusion

We found that water insecurity was highly prevalent among pregnant and postpartum WLWH, and that those with moderate household WI had nearly three times the odds of attaining below average ART adherence scores compared to those with low household WI scores. The findings suggest the importance of prioritizing WI in the context of HIV/AIDS care provision, especially among WLWH. These findings are critical to inform service and policy planning decisions among governmental and other stakeholders. Stakeholders should focus on expanding water supply to communities and homes without water access in the country. Public agencies responsible for water supply should maintain consistent supply the various homes. Finally, government should provide subsidy to people living with HIV who could not afford to access clean water.

## Supporting information

S1 Table Parameters used to estimate household food insecurity and water insecurity. (DOCX)

S2 Table Bivariate analysis of factors associated with having a below average ART adherence score (N = 176). (DOCX)

S1 Checklist Inclusivity in global research. (DOCX)
