# Supplementary material for: Association between water insecurity and antiretroviral therapy adherence among pregnant and postpartum women in Greater Accra region of Ghana
Source: PLOS Glob Public Health. 2024 Jan 8;4(1):e0002747. doi: 10.1371/journal.pgph.0002747 (PMC10773961; doi:10.1371/journal.pgph.0002747)
Supplement: S1 Table — (DOCX) [file pgph.0002747.s001.docx]

S1 Table: Parameters used to estimate household food insecurity and water insecurity

| **#** | **Parameter** |
| --- | --- |
| **Household food insecurity** | |
| 1 | Did you worry that you would not have enough food |
| 2 | Were you not able to eat the kinds of foods you preferred because of a lack of resources? |
| 3 | Did you or any household member have to eat a limited variety of foods due to a lack of resources? |
| 4 | Did you or any household member have to eat some foods that you really did not want to eat because of a lack of resources to obtain other types of food? |
| 5 | Did you or any household member have to eat a smaller meal than you felt you needed because there was not enough food? |
| 6 | Did you eat fewer meals in a day because there was not enough food? |
| 7 | Was there ever no food to eat of any kind in your household because of lack of resources to get food? |
| 8 | Did you or any household member go to sleep at night hungry because there was not enough food? |
| 9 | Did you or any household member go a whole day and night without eating anything because there was not enough food? |
| **Household water insecurity** | |
| 1 | How frequently did you or anyone in your household worry you would not have enough water for all of your household needs? |
| 2 | How frequently has your main water source been interrupted or limited (eg, water pressure, less water than expected, river dried up)? |
| 3 | How frequently have problems with water meant that clothes could not be washed? |
| 4 | How frequently have you or anyone in your household had to change schedules or plans due to problems with your water situation? (Activities that may have been interrupted include caring for others, doing household chores, agricultural work, income-generating activities, etc.) |
| 5 | How frequently have you or anyone in your household had to change what was being eaten because there were problems with water (eg, for washing foods, cooking, etc.)? |
| 6 | How frequently have you or anyone in your household had to go without washing hands after dirty activities (eg, defecating or changing diapers, cleaning animal dung) because of problems with water? |
| 7 | How frequently have you or anyone in your household had to go without washing their body because of problems with water (eg, not enough water, dirty, unsafe)? |
| 8 | How frequently has there not been as much water to drink as you would like for you or anyone in your household? |
| 9 | How frequently did you or anyone in your household feel angry about your water situation? |
| 10 | How frequently have you or anyone in your household gone to sleep thirsty because there wasn't any water to drink? |
| 11 | How frequently has there been no usable or drinkable water whatsoever in your household? |
| 12 | How frequently have problems with water caused you or anyone in your household to feel  shamed/excluded/stigmatised? |
