# Supplementary material for: Association between water insecurity and antiretroviral therapy adherence among pregnant and postpartum women in Greater Accra region of Ghana
Source: PLOS Glob Public Health. 2024 Jan 8;4(1):e0002747. doi: 10.1371/journal.pgph.0002747 (PMC10773961; doi:10.1371/journal.pgph.0002747)
Supplement: S2 Table — (DOCX) [file pgph.0002747.s002.docx]

**S2 Table: Bivariate analysis of factors associated with having a below average ART adherence score (N=176)**

| **Characteristics** | **Category** | ART adherence score | | |
| --- | --- | --- | --- | --- |
|  |  | **Good (n=117)** | **Poor (n=59)** | **p** |
| Place of residence | Peri-urban/Rural | 29 (24.8) | 7 (11.9) | 0.045 |
|  | Urban | 88 (75.2) | 52 (88.1) |  |
| Age (n=174) | <30 years | 29 (25.0) | 25 (43.1) | 0.043 |
|  | 30-34 | 48 (41.4) | 16 (27.6) |  |
|  | >=35 years | 39 (33.6) | 17 (29.3) |  |
| Marital status | Married/separated | 66 (56.4) | 32 (54.2) | 0.920 |
|  | In a relationship, living/not with a partner | 46 (39.3) | 25 (42.4) |  |
|  | Single, never married, no current partner | 5 (4.3) | 2 (3.4) |  |
| Partner’s HIV status known | No | 35 (29.9) | 30 (50.9) | 0.007 |
|  | Yes | 82 (79.1) | 29 (49.1) |  |
| Have rival/co-wife or co-wives | Yes | 8 (7.1) | 10 (17.5) | 0.038 |
|  | No | 104 (92.8) | 47 (82.5) |  |
| Number of children (n=173) | None | 10 (8.8) | 9 (15.3) | 0.388 |
|  | 1-2 children | 54 (47.4) | 28 (47.4) |  |
|  | 3 or more children | 50 (43.8) | 22 (37.3) |  |
| Religion | Protestant/catholic | 24 (20.5) | 9 (15.3) | 0.833 |
|  | Pentecostal | 36 (30.8) | 18 (30.5) |  |
|  | Charismatic | 37 (31.6) | 20 (33.9) |  |
|  | Others (Adventist/Muslim/..) | 20 (17.1) | 12 (20.3) |  |
| Education status | Less than primary | 35 (29.9) | 12 (20.3) | 0.136 |
|  | Completed primary | 33 (28.2) | 27 (45.8) |  |
|  | Completed O level | 30 (25.6) | 12 (20.3) |  |
|  | Completed A level and above | 19 (16.2) | 8 (13.6) |  |
| Fuel type mainly used | LPG or natural gas | 68 (58.1) | 44 (74.6) | 0.032 |
|  | Charcoal/wood | 49 (41.9) | 15 (25.4) |  |
| Household Food Insecurity score | Low food insecurity | 44 (37.6) | 19 (32.2) | 0.350 |
|  | Moderate food insecurity | 40 (34.2) | 17 (28.8) |  |
|  | High food insecurity | 33 (28.2) | 23 (39.0) |  |
| Household Water Insecurity score | Low water insecurity | 54 (46.2) | 13 (22.0) | 0.007 |
|  | Moderate water insecurity | 30 (25.6) | 24 (40.7) |  |
|  | High water insecurity | 33 (28.2) | 22 (37.3) |  |
